# Metastability and Entropy Peaks in Antagonistic Multiplex Consensus Dynamics

**DOI:** 10.3390/e28080857

**Published:** 2026-08-01

**Authors:** Jack C. M. Hughes, Anna Kusmartseva, Glenn Muschert, Herbert F. Jelinek, Fedor V. Kusmartsev

**Affiliations:** 1 Department of Public Health and Epidemiology, College of Medicine and Health Sciences, Khalifa University, Abu Dhabi P.O. Box 127788, United Arab Emirates; glenn.muschert@ku.ac.ae; 2 Department of Physics, College of Engineering and Physical Sciences, Khalifa University, Abu Dhabi P.O. Box 127788, United Arab Emirates; fedor.kusmartsev@ku.ac.ae; 3 Department of Physics, Loughborough University, Loughborough LE11 3TU, UK; a.kusmartseva@lboro.ac.uk; 4 Healthcare Engineering Innovation Group (HEIG), Khalifa University, Abu Dhabi P.O. Box 127788, United Arab Emirates; herbert.jelinek@ku.ac.ae; 5 Department of Medical Sciences, College of Medicine and Health Sciences, Khalifa University, Abu Dhabi P.O. Box 127788, United Arab Emirates

**Keywords:** opinion dynamics, multiplex networks, metastability, shannon entropy, polarization, nonlinear dynamics

## Abstract

Modern societies comprise overlapping communities whose opinions evolve on strongly interacting networks that are often mutually antagonistic. We introduce a minimal antagonistic multiplex consensus model in which each layer follows intra-layer majority-rule dynamics, while inter-layer interactions are inhibitory. A mean-field analysis shows that antagonistic coupling destabilizes the balanced state through an antisymmetric mode and favors two polarized absorbing states with opposite magnetization in the two layers. Network-averaged simulations confirm that small fluctuations near equal initial support determine which polarized state is ultimately reached: trajectories exhibit metastable delay, long convergence times, and a localized peak in the Shannon entropy of outcomes. A finite-size analysis with independent network realizations and bootstrap uncertainty estimates shows that the high-entropy interval narrows as Δr $∼N^{-\gamma_{\mathrm{eff}}}$, with $\gamma_{\mathrm{eff}}=0.513$ and a 95% bootstrap confidence interval [0.489,0.526], consistent with finite-size sharpening controlled by fluctuations in the initial imbalance. We also perform network topology checks and find that the qualitatively antagonistic mechanism persists beyond random-regular graphs. As an illustrative empirical application, we analyze county-level results from the 2024 U.S. presidential election. The vote-share and entropy landscapes separate low-entropy partisan strongholds from higher-entropy competitive counties. Our results suggest that antagonistic multiplex coupling provides a simple mechanism by which polarized attractors and localized outcome uncertainty can arise together.

## 1. Introduction

Societies are increasingly shaped by interactions on complex social networks, where information, opinions, and behaviors spread through heterogeneous patterns of influence that are far from homogeneous [1]. Digital platforms, partisan media ecosystems, and geographically rooted communities generate overlapping and often antagonistic environments of social influence: in such settings, individuals are simultaneously embedded in multiple social circles that promote competing narratives [2,3]. The resulting tension between local conformity and cross-group antagonism is widely believed to underlie the persistent polarization and unpredictability observed in contemporary politics and public discourse. To this effect, political applications of statistical physics were developed by Galam [4], while the effect of exposure to ideologically diverse information on social platforms was quantified by Bakshy et al. [5]. More recently, mechanisms for understanding and mitigating polarization have been explored [6,7], together with the modeling of the emergence of echo chambers and political fragmentation [8,9].

Classical opinion formation has often been studied on a single interaction layer. Spatial conflict and voter-model dynamics were introduced by Clifford and Sudbury [10] and by Holley and Liggett [11]. Majority-rule opinion dynamics were developed by Galam [12] and by Krapivsky and Redner [13]; related kinetic approaches are reviewed in Ref. [14]. These models exhibit consensus, coexistence, or coarsening depending on the update rule, topology, and noise. The theory of interacting particle systems provides rigorous foundations for many of these processes [15], and voter-like critical coarsening was analyzed by Dornic et al. [16]. However, real societies are better described as multiplex systems, where the same individuals participate in several distinct but overlapping interaction layers, such as face-to-face communities, online platforms, and professional or ideological networks. Diffusion dynamics on multiplex networks were shown by Gómez et al. to differ qualitatively from single-layer dynamics [17], and Diakonova et al. demonstrated that multilayer structures can alter voter-model behavior [18]. General frameworks for multilayer networks were developed by Kivelä et al. [19] and Boccaletti et al. [20]. More specialized results regarding the entropy and overlap of multiplex ensembles were studied by Bianconi [21], and growth processes in multiplex networks were developed in detail by Nicosia et al. [22].

A key feature of many real systems is that the interaction between communities is not only cooperative but actively antagonistic. Rival political parties, competing media ecosystems, and opposed ideological groups often seek not only to strengthen support within their own communities, but also to erode the influence of competing ones. Existing multilayer models often implement cooperative or neutral coupling between layers, while disagreement in single-layer models is frequently introduced through contrarian agents, zealots, noise, or fixed heterogeneity. These mechanisms are important, but they do not directly model frustrated competition between entire social networks that are themselves internally cohesive yet mutually inhibiting. The idea of frustration is related to the structural-balance theory introduced by Heider [23] and formalized by Cartwright and Harary [24]; energy landscapes for social balance were later studied by Marvel, Strogatz, and Kleinberg [25]. Nevertheless, the mechanisms by which antagonistic multiplex structure can produce robust polarization, metastability, and localized unpredictability remain only partially understood.

The novelty of the present work is consequently the combination of majority-rule consensus within each layer with explicitly antagonistic coupling *between layers*. Explicitly, we introduce and analyze a minimal model of competing social networks in which each layer is internally consensus-seeking, whereas the two layers mutually inhibit one another. This creates a frustrated dynamical system in which local conformity and cross-layer competition cannot be simultaneously satisfied. At the microscopic level, each agent carries a pair of binary states, one in each layer, and updates synchronously according to a local majority rule modified by inhibitory inter-layer coupling. Despite its simplicity, this rule produces a nontrivial competition between two polarized final states. A regularized mean-field analysis shows that the balanced state at approximately equal initial support is destabilized by an antisymmetric mode, corresponding to perturbations that increase support in one layer while decreasing support in the other. The resulting polarized states, in which the two layers have opposite magnetization, are absorbing states of the underlying sign-rule dynamics.

We perform numerical simulations to confirm this dynamical picture. When the initial support ratio is far from r=1/2, the system rapidly converges to the polarized state favored by the initial bias. Near r=1/2, however, trajectories remain close to the balanced state for long times before one of the two polarized outcomes is selected. This produces a peak in the convergence time and a localized maximum in the Shannon entropy of outcomes. The entropy peak quantifies the fact that, in this narrow region, small differences in initial conditions, tie-breaking, or network realization can determine which polarized state is ultimately reached.

To quantify the finite-size behavior, we average the main numerical observables over independent network realizations, as well as over dynamical runs. The width of the high-entropy interval is measured by the full width at half maximum Δr of the entropy profile. Bootstrap resampling of network-level entropy curves gives $\Delta r∼N^{-\gamma_{\mathrm{eff}}},\gamma_{\mathrm{eff}}=0.513$ with a 95% bootstrap confidence interval [0.489,0.526]. We interpret this as an effective finite-size exponent for the random-regular topology and parameter range studied. Its proximity to 1/2 is consistent with the natural scale of finite-size fluctuations in the initial support imbalance, which shrink as ∼$N^{-1/2}$. Additionally, checks on alternative graph ensembles further indicate that the qualitative competition between polarized outcomes is a natural outcome of the model, although the quantitative entropy width and convergence times are topology-dependent (especially when restricted to the small-world case, which we find does not conform with this picture on the considered time-scales).

To illustrate the diagnostic relevance of this mechanism in real-world settings, we analyze county-level results from the 2024 U.S. presidential election. The empirical distribution of vote shares displays bimodality, while the associated entropy landscape separates low-entropy partisan strongholds from high-entropy competitive counties. We present this comparison as a qualitative diagnostic analogy. The empirical analysis asks whether the data display the same statistical signatures predicted by the model: polarized low-entropy basins and a localized high-entropy competitive region, which we find is the case.

The paper is organized as follows. Section 2 defines the antagonistic multiplex model, the discrete-time update rule, and the initial conditions. Section 3 develops the regularized mean-field analysis, identifies the unstable balanced state, and describes the polarized absorbing states and characteristic modes. Section 4 presents the network-averaged simulations, entropy peak, finite-size scaling with bootstrap uncertainty, and topology checks. Section 5 applies the entropy diagnostics to U.S. county-level election data. Section 6 and Section 7 discuss limitations, extensions, and conclusions.

## 2. Model and Dynamics

The antagonistic coupling between layers introduces frustration analogous to that found in spin systems with competing interactions. Spin-glass frustration has been reviewed by Young [26], by Nishimori [27], and by Mézard, Parisi, and Virasoro [28]. Critical phenomena in complex networks were reviewed by Dorogovtsev, Goltsev, and Mendes [29], and related binary-network models of opinion and decision dynamics have been considered by Kusmartsev and Kürten [30,31].

We consider a two-layer multiplex network with layers *A* and *B*, each containing *N* nodes. The same individual *i* appears in both layers and is assigned a pair of binary variables

(1) $$\left(s_{i}^{A}\left(t\right),s_{i}^{B}\left(t\right)\right),\;s_{i}^{A}\left(t\right),s_{i}^{B}\left(t\right)\in \left\{+1,-1\right\}.$$

The value +1 denotes support for the corresponding layer’s opinion, ideology, or state, while −1 denotes opposition. Each layer has its own intra-layer graph, with neighborhoods $N_{A}\left(i\right)$ and $N_{B}\left(i\right)$. The inter-layer coupling is one-to-one: node *i* in layer *A* interacts only with its counterpart *i* in layer *B*.

In the baseline simulations, both layers are independent random 3-regular graphs, so that every node has exactly three intra-layer neighbors. This topology is used as a controlled minimal case, since it removes degree heterogeneity and isolates the effect of antagonistic inter-layer coupling. The same update rule can also be applied to other graph ensembles, such as (for instance) Erdös–Rényi, small-world and scale-free networks; these will be considered later as limited topology checks against the dynamics of the model.

Opinion dynamics proceed in discrete time and are updated synchronously. Within each layer, agents tend to align with the local majority of their intra-layer neighbors. Between layers, the coupling is antagonistic: support in one layer suppresses support in the other. The microscopic update rules are

(2) $$s_{i}^{A}\left(t+1\right)=\mathrm{sign}\left(\sum_{j\in N_{A}\left(i\right)}s_{j}^{A}\left(t\right)+J\;s_{i}^{B}\left(t\right)\right),$$

(3) $$s_{i}^{B}\left(t+1\right)=\mathrm{sign}\left(\sum_{j\in N_{B}\left(i\right)}s_{j}^{B}\left(t\right)+J\;s_{i}^{A}\left(t\right)\right),$$

where *J* is the inter-layer coupling strength. In this work, the main focus is the antagonistic case J<0. When the argument of the sign function is zero, the tie is resolved randomly by assigning +1 or −1 with equal probability.

The initial condition is controlled by a scalar parameter r∈[0,1], representing the initial support bias toward layer *A*. For each node *i*, the two layer states are sampled independently according to

(4) $$P\left(s_{i}^{A}\left(0\right)=+1\right)=r,\;P\left(s_{i}^{A}\left(0\right)=-1\right)=1-r$$

and

(5) $$P\left(s_{i}^{B}\left(0\right)=+1\right)=1-r,\;P\left(s_{i}^{B}\left(0\right)=-1\right)=r.$$

The balanced ensemble corresponds to r=1/2. Values r>1/2 initially favor layer *A*, while values r<1/2 initially favor layer *B*.

The use of a discrete-time synchronous update is appropriate for binary majority-rule dynamics, where each step represents one round of collective opinion revision. This differs from continuous-time network dynamical systems, in which real-valued node states evolve gradually according to differential equations. Continuous models are natural for diffusion, reaction–diffusion, and Turing-type instabilities [32], whereas the present model is designed to study abrupt binary switching, competition between polarized outcomes, and metastability under antagonistic multiplex coupling.

## 3. Analytical Framework

To understand how large-scale patterns arise from the microscopic rules in Equations (2) and (3), we analyze the evolution of the average opinions (or what amounts to magnetizations) in the two layers. For networks *A* and *B*, we define

(6) $$m_{A}\left(t\right)=\frac{1}{N}\sum_{i=1}^{N}s_{i}^{A}\left(t\right),\;m_{B}\left(t\right)=\frac{1}{N}\sum_{i=1}^{N}s_{i}^{B}\left(t\right),$$

where $m_{A},m_{B}\in \left[-1,1\right]$ measure the net support for each network at time *t*. Thus, the expected initial magnetizations are

(7) $$E\left[m_{A}\left(0\right)\right]=2r-1,\;E\left[m_{B}\left(0\right)\right]=1-2r.$$

### 3.1. Mean-Field Approximation and Smooth Regularization

In a mean-field approximation, we neglect correlations between neighboring spins and assume that each node in a given layer feels the same effective field. For a *k*-regular network, this field is

(8) $$h_{A}\left(t\right)=k\;m_{A}\left(t\right)+J\;m_{B}\left(t\right),\;h_{B}\left(t\right)=k\;m_{B}\left(t\right)+J\;m_{A}\left(t\right),$$

where *k* is the intra-layer degree (here k=3) and *J* is the inter-layer coupling introduced in the Model section. The majority-rule dynamics then update the average opinions according to the signs of these effective fields.

At a coarse-grained level, the mean-field dynamics can be written as

(9) $$m_{A}\left(t+1\right)=F\;\left(h_{A}\left(t\right)\right),\;m_{B}\left(t+1\right)=F\;\left(h_{B}\left(t\right)\right),$$

where F(x) is an odd, monotonically increasing response function with F(0)=0. In the microscopic simulations, the update rule is the discontinuous sign function appearing in (2) and (3). For the stability analysis, however, it is useful to regard this sign rule as the sharp-response limit of a smooth regularization. A convenient explicit family is

(10) $$F_{\beta }\left(x\right)=\tanh\left(\beta x\right)$$

where β>0 controls the steepness of the response. For finite β, $F_{\beta }^{\prime }\left(0\right)=\beta$ is well defined, so the linear stability calculation is meaningful. In the limit β→∞, $F_{\beta }\left(x\right)\to \mathrm{sign}\left(x\right)$ for x≠0, recovering the majority-rule dynamics used in the simulations. Thus, we may think of the smooth-response map as a regularized mean-field description of the same binary update mechanism.

### 3.2. Fixed Points

Fixed points of the mean-field map satisfy

(11) $$m_{A}^{*}=F\;\left(k\;m_{A}^{*}+J\;m_{B}^{*}\right),\;m_{B}^{*}=F\;\left(k\;m_{B}^{*}+J\;m_{A}^{*}\right).$$

Since the equations are symmetric under exchange of the two layers, it is useful to consider symmetric and antisymmetric combinations.

For symmetric states, $m_{A}^{*}=m_{B}^{*}=m^{*}$, giving

(12) $$m^{*}=F\;\left(\left(k+J\right)\;m^{*}\right).$$

These states correspond to both layers having the same average orientation. They are naturally favored by cooperative coupling J>0.

For antisymmetric states, $m_{A}^{*}=-m_{B}^{*}=m^{*}$, giving

(13) $$m^{*}=F\;\left(\left(k-J\right)\;m^{*}\right).$$

For antagonistic coupling J<0, the effective gain k−J=k+|J| is enhanced, so the antisymmetric branch is the dynamically relevant polarized branch. These states correspond to one layer being predominantly supported while the other is predominantly opposed. These antisymmetric consensus states form the basis of the simulations described below.

### 3.3. Linear Stability and the Unstable Balanced State

To determine the stability of these states, we linearize the mean-field map around a fixed point $\left(m_{A}^{*},m_{B}^{*}\right)$. Writing

(14) $$m_{A}\left(t\right)=m_{A}^{*}+\delta m_{A}\left(t\right),\;m_{B}\left(t\right)=m_{B}^{*}+\delta m_{B}\left(t\right),$$

one obtains

(15) $$\left(\begin{array}{c} \delta m_{A}\left(t+1\right)\\ \delta m_{B}\left(t+1\right) \end{array}\right)=J_{*}\left(\begin{array}{c} \delta m_{A}\left(t\right)\\ \delta m_{B}\left(t\right) \end{array}\right)$$

where

(16) $$J_{*}=\left(\begin{array}{cc} F^{\prime }\left(h_{A}^{*}\right)k & F^{\prime }\left(h_{A}^{*}\right)J\\ F^{\prime }\left(h_{B}^{*}\right)J & F^{\prime }\left(h_{B}^{*}\right)k \end{array}\right).$$

At the balanced state $\left(m_{A}^{*},m_{B}^{*}\right)=\left(0,0\right)$, one has $h_{A}^{*}=h_{B}^{*}=0$. The eigenvectors are the symmetric mode (1,1) and the antisymmetric mode (1,−1), with eigenvalues (respectively)

(17) $$\lambda_{+}=F^{\prime }\left(0\right)\left(k+J\right),\;\lambda_{-}=F^{\prime }\left(0\right)\left(k-J\right).$$

The balanced state is linearly stable only if

(18) $$|\lambda_{+}\left|\;<1,\;\right|\lambda_{-}|\;<1.$$

For the regularization $F_{\beta }\left(x\right)=\tanh\left(\beta x\right)$, this becomes

(19) $$\lambda_{+}=\beta \left(k+J\right),\;\lambda_{-}=\beta \left(k-J\right).$$

In the antagonistic case J<0, the antisymmetric eigenvalue is enhanced:

(20) $$\lambda_{-}=\beta \left(k-J\right)=\beta \left(k+|J|\right).$$

Thus, perturbations that increase the magnetization in one layer while decreasing it in the other are preferentially amplified. This is the linear mechanism by which the balanced state loses stability toward polarized states with opposite layer magnetizations.

It is worth emphasizing that in the sharp majority-rule limit β→∞, the derivative of the sign function is not defined at zero. The balanced state (in the coarse-grained limit) is therefore maximally sensitive to infinitesimal perturbations, consistent with the sign-rule simulations (see below): small perturbations in either the symmetric or antisymmetric direction are amplified, pushing the system towards one of the polarized consensus attractors described below.

### 3.4. Polarized Absorbing States and Attraction Structure

The linear stability calculation above describes the behavior near the balanced state. At the microscopic sign-rule level, the fully polarized states are absorbing. Consider the *A*-dominated state

(21) $$s_{i}^{A}=+1,\;s_{i}^{B}=-1$$

for all nodes *i*. On a *k*-regular graph and for J<0, the local fields are

(22) $$h_{i}^{A}=k-J=k+|J|>0,\;h_{i}^{B}=J-k=-\left(k+|J|\right)<0.$$

Therefore, the sign update leaves every spin unchanged. The *B*-dominated state,

(23) $$s_{i}^{A}=-1,\;s_{i}^{B}=+1,$$

is absorbing by symmetry.

These absorbing states are the two polarized outcomes observed in the simulations. The balanced state separates their regions of attraction in the mean-field picture. Near r=1/2, finite-size fluctuations, random tie-breaking, and network realization determine which polarized state is eventually reached, giving rise to long convergence times and large outcome entropy. As the system size *N* increases, fluctuations become smaller, and the unstable fixed point becomes sharper in phase space. Consequently, the high-entropy region in *r* shrinks, and the transition between the two consensus phases becomes increasingly steep.

### 3.5. Finite Network Characteristic Values

The same instability can be expressed at the level of the finite multiplex network. Let $A_{A}$ and $A_{B}$ be the adjacency matrices of the two layers. The signed supra-adjacency matrix associated with the linear part of the update rule is

(24) $$M=\left(\begin{array}{cc} A_{A} & JI\\ JI & A_{B} \end{array}\right).$$

For the regularized response $F_{\beta }\left(x\right)=\tanh\left(\beta x\right)$, perturbations around the balanced state satisfy, to leading order,

(25) δs(t+1)≃βMδs(t).

Thus, the characteristic values of βM determine the early growth or decay of perturbations near the balanced state. If the spectral radius satisfies

(26) ρ(βM)>1,

then the balanced state is linearly unstable in the regularized finite-network description.

For identical *k*-regular layers, the uniform intra-layer mode has eigenvalue *k*. The corresponding symmetric and antisymmetric supra-network modes are (v,v) and (v,−v), with characteristic values

(27) $$\mu_{+}=k+J,\;\mu_{-}=k-J.$$

**Figure 1:**
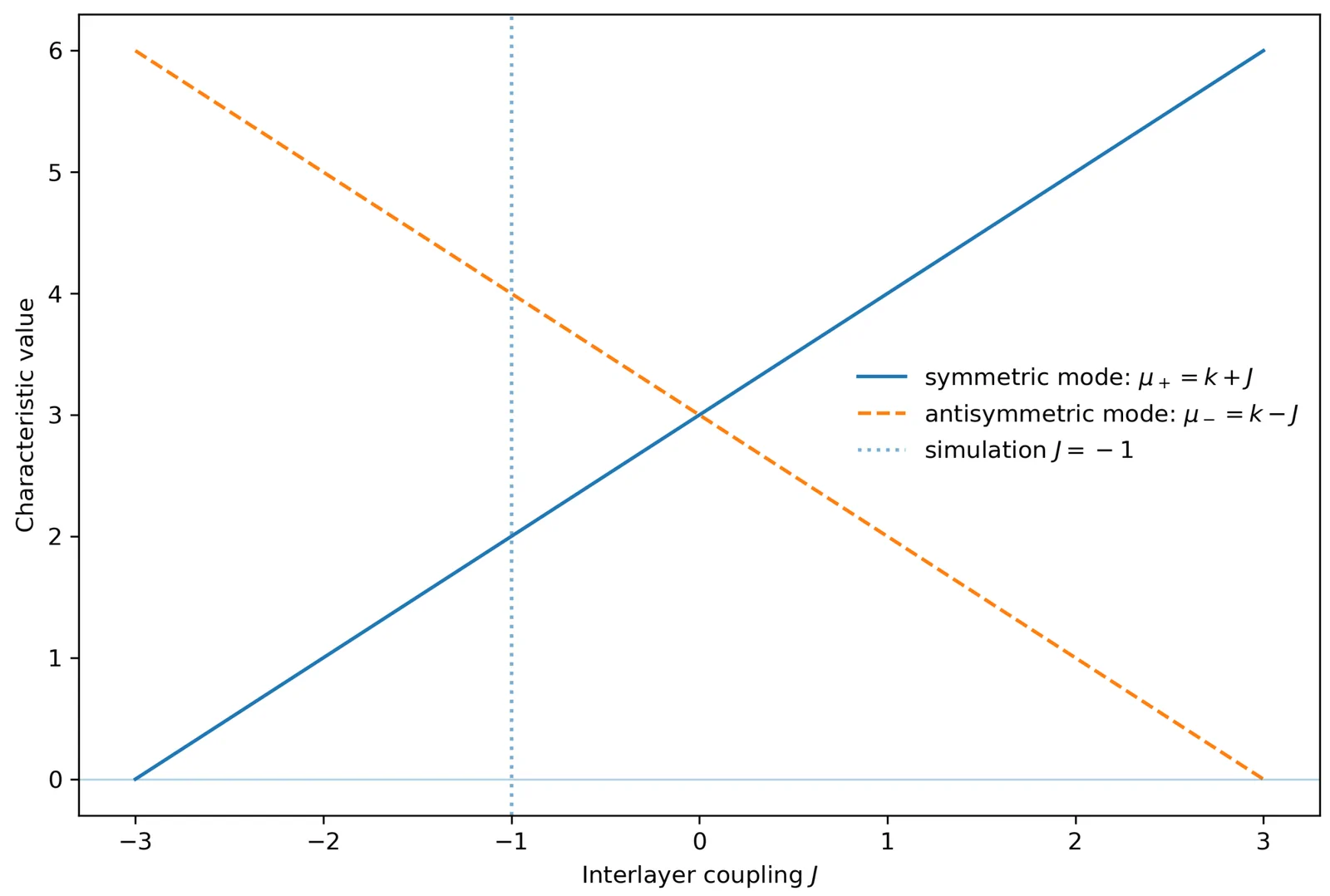
Uniform-mode characteristic values of the signed supra-network matrix for k=3. For identical *k*-regular layers, the symmetric supra-network mode has characteristic value $\mu_{+}=k+J$, while the antisymmetric mode has characteristic value $\mu_{-}=k-J$. For antagonistic coupling J<0, the antisymmetric mode dominates. At the simulation value J=−1, one obtains $\mu_{+}=2$ and $\mu_{-}=4$, indicating preferential amplification of perturbations with opposite signs in the two layers.

For antagonistic coupling J<0, the antisymmetric value $\mu_{-}=k+\left|J\right|$ is larger than the symmetric value $\mu_{+}=k-\left|J\right|$. The dominant instability is therefore the mode in which the two layers move in opposite directions (see Figure 1).

For non-regular graphs, such as the Erdös–Rényi, small-world, and scale-free ensembles considered in the numerical checks below, the same supra-adjacency formulation applies, but the characteristic values depend on the full spectra of $A_{A}$ and $A_{B}$. This is why, as we shall see, the qualitative antagonistic mechanism can persist across graph classes while the quantitative transition width, relaxation time, and metastable fraction remain topology-dependent.

## 4. Numerical Results

In this section, we investigate the collective dynamics of the antagonistically coupled multiplex system defined by Equations (2) and (3). We first describe the simulation protocol, then examine the emergence of metastability and polarized consensus, quantify the associated entropy peak, perform a limited topology robustness check, and finally compare the antagonistic case with cooperative inter-layer coupling for completeness.

### 4.1. Simulation Setup and Uncertainty Estimation

The baseline simulations use independent random 3-regular graphs for the two layers, where we will average over both structural and dynamical disorder. For each system size *N*, we generate *M* independent multiplex network realizations. Each multiplex realization m=1,…,M consists of two independently generated random 3-regular graphs, one for layer *A* and one for layer *B*, together with the one-to-one inter-layer coupling between corresponding nodes.

For each network realization *m* and each initial support ratio *r*, we perform *R* independent dynamical runs. In each run, the initial states are sampled independently according to the probability law defined in Section 2, and stochastic tie-breaking events are sampled independently. The dynamics are evolved synchronously according to (2) and (3) until either a time-independent configuration is reached or a maximum time $T_{\max}$ is exceeded. The convergence time τ is defined as the first time step at which no spin changes state between successive updates.

Each final state is classified as *A*-dominated, *B*-dominated, or non-consensus/metastable. We call a run *A*-dominated if

(28) $$m_{A}^{f}\geq m_{\mathrm{tol}},\;m_{B}^{f}\leq -m_{\mathrm{tol}},$$

*B*-dominated if

(29) $$m_{A}^{f}\leq -m_{\mathrm{tol}},\;m_{B}^{f}\geq m_{\mathrm{tol}},$$

and non-consensus/metastable otherwise. In the simulations below, we use $m_{\mathrm{tol}}=0.90$.

For each network realization *m*, we compute the network-level outcome probabilities

(30) $$P_{A}^{\left(m\right)}\left(r\right),\;P_{B}^{\left(m\right)}\left(r\right),\;P_{\mathrm{meta}}^{\left(m\right)}\left(r\right),$$

from the *R* dynamical runs. The corresponding network-level Shannon entropy is

(31) $$S^{\left(m\right)}\left(r\right)=-\sum_{\alpha \in \{A,B,\mathrm{meta}\}}P_{\alpha }^{\left(m\right)}\left(r\right)\ln P_{\alpha }^{\left(m\right)}\left(r\right).$$

The plotted entropy curve is the average over network realizations,

(32) $$\bar{S}\left(r\right)=\frac{1}{M}\sum_{m=1}^{M}S^{\left(m\right)}\left(r\right),$$

and uncertainty bands denote the standard error across the *M* independent network-level curves. The same averaging procedure is used for $P_{A}\left(r\right)$, $P_{B}\left(r\right)$, $P_{\mathrm{meta}}\left(r\right)$, and 〈τ〉(r).

In the finite-size analysis reported below, we used k=3, J=−1, r∈[0.44,0.56] with 121 equally spaced values of *r*, M=25 independent network realizations, R=100 dynamical runs per network realization, and $T_{\max}=150$. Thus, each value of *r* is estimated from MR=2500 runs for each system size. The system sizes considered were

(33) N=400,800,1200,1500,2000,2500,3000.

### 4.2. Representative Trajectories and Polarized Consensus

**Figure 2:**
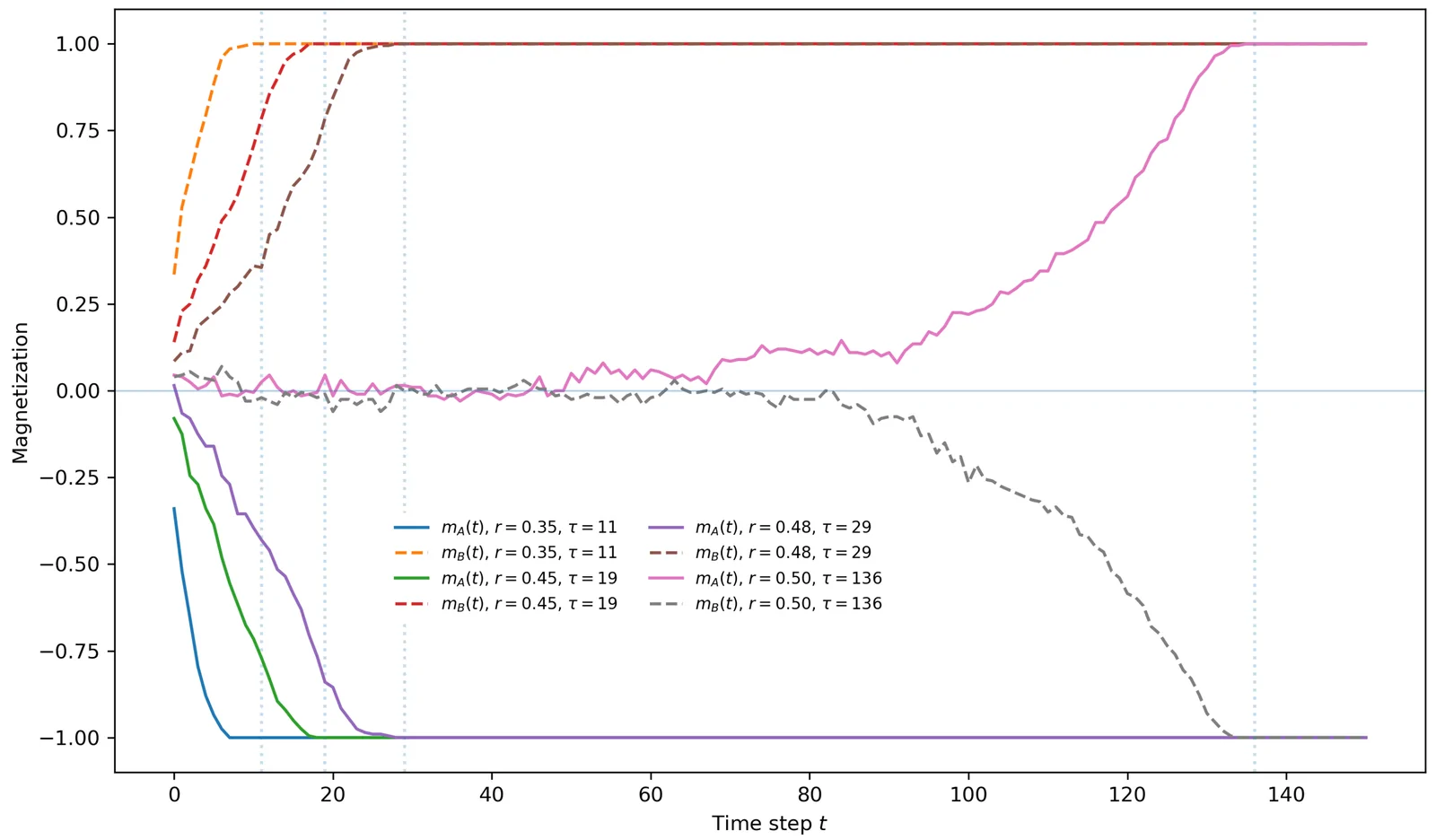
Representative time evolution of the layer magnetizations in antagonistically coupled multiplex networks. All trajectories are plotted over the same time window for N=400. Once a trajectory reaches a fixed point, its final magnetization is continued as a horizontal plateau. Strongly biased initial conditions converge rapidly to polarized consensus states, while trajectories with *r* close to 1/2 remain near the balanced state for longer times before collapsing into one of the two polarized attractors. The different plateau onset times correspond to different convergence times τ.

For antagonistic coupling J<0, the long-time dynamics typically approach one of two polarized states: an A-dominated state with $m_{A}^{f}≃+1$ and $m_{B}^{f}≃-1$, or a B-dominated state with $m_{A}^{f}≃-1$ and $m_{B}^{f}≃+1$. Figure 2 shows representative trajectories of $m_{A}\left(t\right)$ and $m_{B}\left(t\right)$ for different initial support ratios.

When the initial support ratio is far from r=1/2, the initial bias rapidly selects one of the two polarized states. Near r=1/2, trajectories remain close to the balanced configuration $\left(m_{A},m_{B}\right)≃\left(0,0\right)$ for longer times before eventually reaching one of the polarized outcomes. This delayed relaxation is the finite-network manifestation of the instability of the balanced state discussed in Section 3.

### 4.3. Outcome Probabilities and Entropy Peak

To quantify the competition between the two polarized outcomes, we compute the probability $P_{A}\left(r\right)$ that a realization ends in an A-dominated state, the probability $P_{B}\left(r\right)$ that it ends in a B-dominated state, and the probability $P_{\mathrm{meta}}\left(r\right)$ that it remains non-consensus or metastable up to $T_{\max}$. By construction,

(34) $$P_{A}\left(r\right)+P_{B}\left(r\right)+P_{\mathrm{meta}}\left(r\right)=1.$$

**Figure 3:**
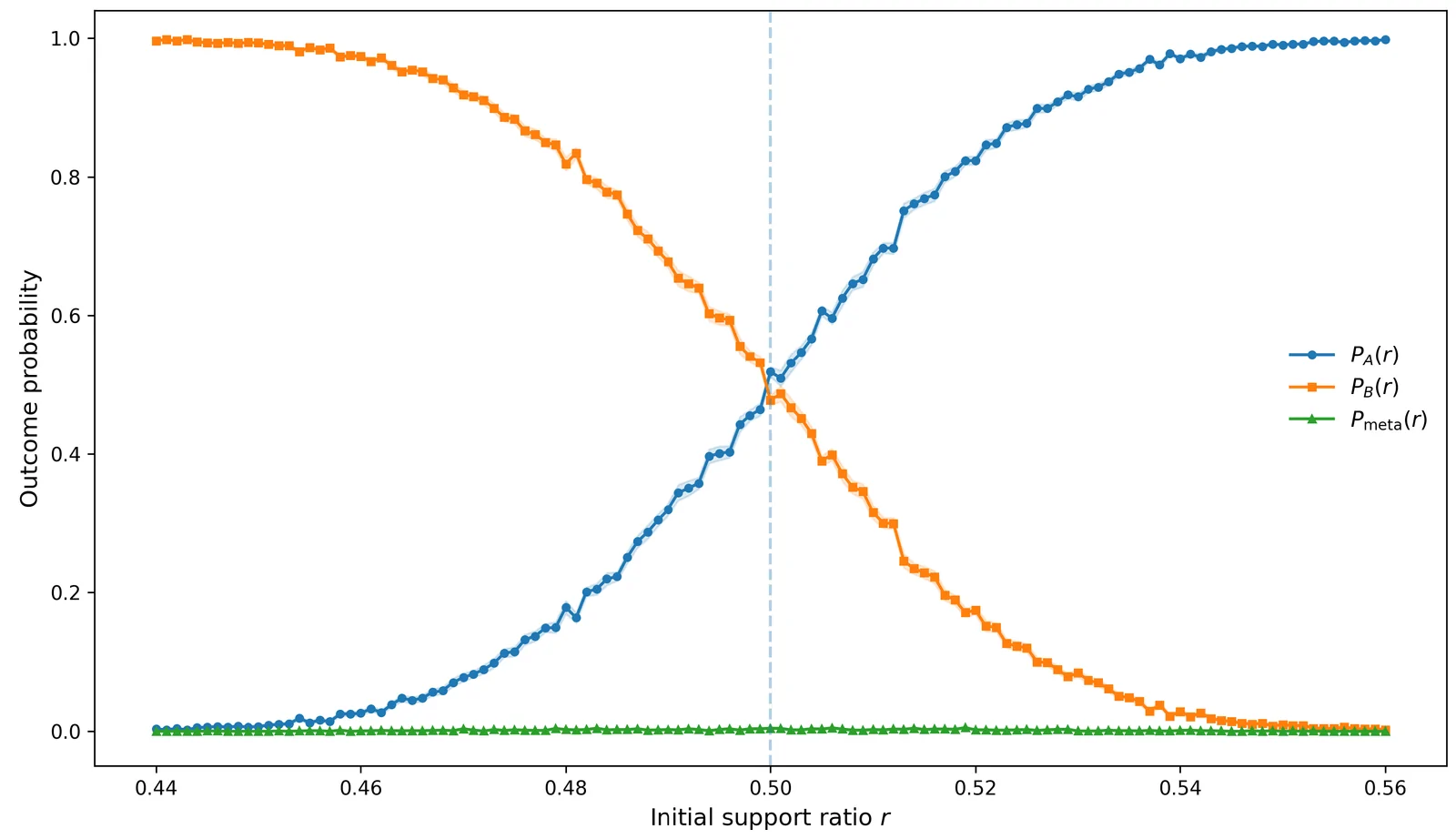
Network-averaged outcome probabilities for N=400 as a function of the initial support ratio *r*. The curves show the probabilities of A-dominated, B-dominated, and non-consensus/metastable outcomes, averaged over independent network realizations (with the standard-error band shown) and dynamical runs. The sharp crossover near r≃1/2 reflects the competition between the two polarized final states.

As shown in Figure 3, the probability $P_{A}\left(r\right)$ changes sharply near r≃1/2. For r<1/2, most realizations reach the B-dominated state; for r>1/2, most realizations reach the A-dominated state. The transition region corresponds to initial conditions close to the unstable balanced state, where finite-size fluctuations, network realization, and stochastic tie-breaking can affect which polarized outcome is reached. For confidently large $T_{\max}$ and *N*, $P_{\mathrm{meta}}\left(r\right)$ becomes negligible, and the system almost always ends in one of the two consensus phases.

**Figure 4:**
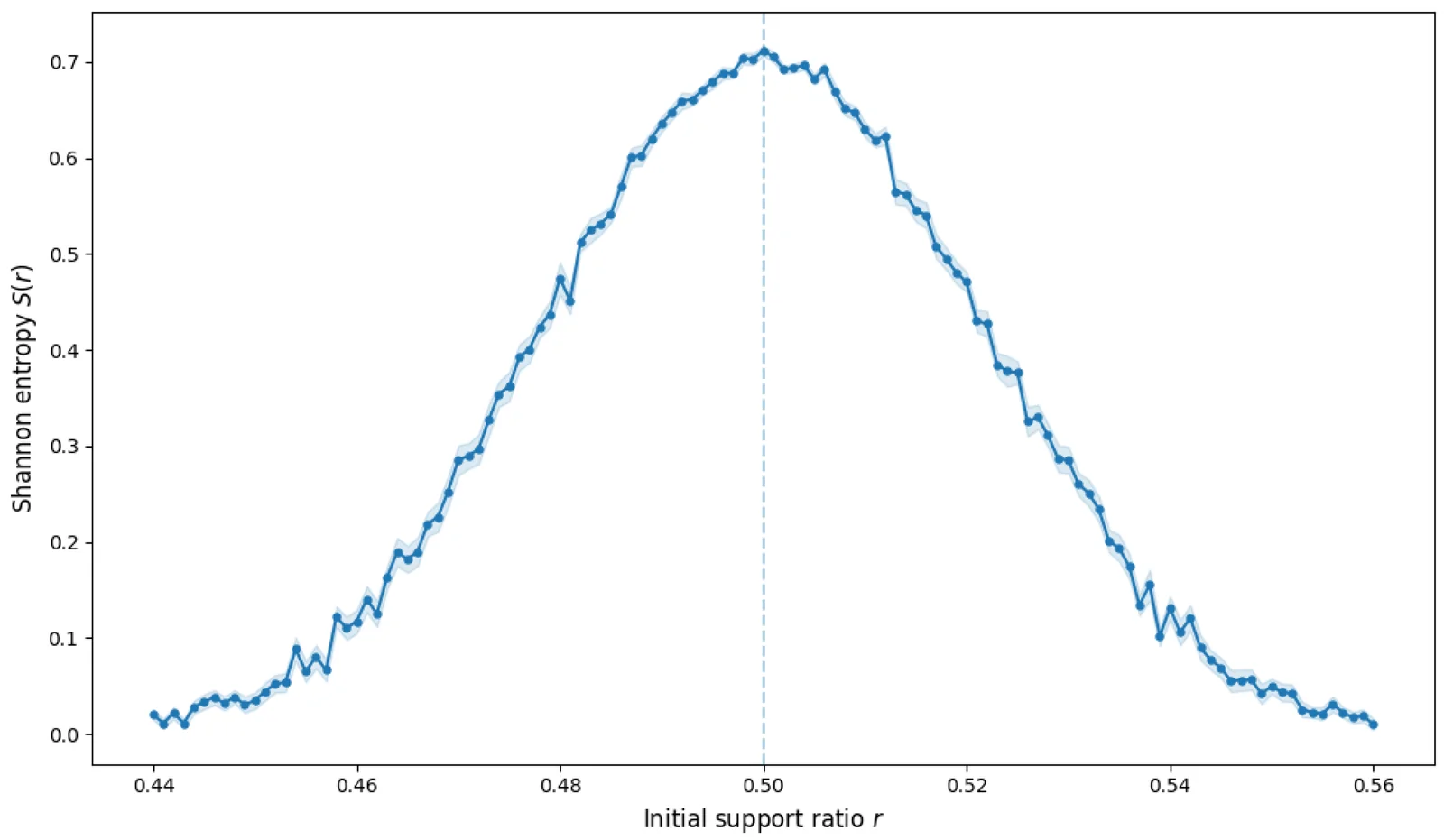
Network-averaged Shannon entropy of outcomes for N=400 as a function of the initial support ratio *r*. For each *r*, the entropy $S\left(r\right)=-\sum_{\alpha }p_{\alpha }\left(r\right)\ln p_{\alpha }\left(r\right)$ is computed from the empirical probabilities of A-dominated, B-dominated, and non-consensus/metastable outcomes. Curves show means over independent network realizations, and shaded regions indicate standard errors across network realizations. The entropy peak remains localized near r≃1/2, showing that outcome uncertainty is concentrated near the boundary between the two polarized outcomes.

The metastable behavior near $r_{c}$ implies that the final state is highly sensitive to microscopic fluctuations in this regime. The outcome entropy

(35) $$S\left(r\right)=-\sum_{\alpha \in \{A,B,\mathrm{meta}\}}p_{\alpha }\left(r\right)\ln p_{\alpha }\left(r\right)$$

provides a scalar measure of this uncertainty. Far from r=1/2, one outcome dominates and S(r)≃0. Near r=1/2, the distribution over outcome classes broadens, producing a localized entropy peak. S(r) is computed for each independent network realization and then averaged across networks; the shaded bands in Figure 4 show the corresponding standard errors.

### 4.4. Finite-Size Sharpening of the Entropy Peak

To quantify the narrowing of the high-entropy region, we define Δr as the full width at half maximum (FWHM) of the entropy profile. Explicitly, for each system size *N* let ${\bar{S}}_{N}\left(r\right)$ denote the entropy curve averaged over independent network realizations, and let

(36) $$S_{\max}\left(N\right)=\max_{r}{\bar{S}}_{N}\left(r\right).$$

The half-maximum level is $S_{\max}\left(N\right)/2$. We determine the left and right crossing points of ${\bar{S}}_{N}\left(r\right)$ with this level by linear interpolation between neighboring *r*-grid points and define

(37) $$\Delta r\left(N\right)=r_{\mathrm{right}}\left(N\right)-r_{\mathrm{left}}\left(N\right).$$

Thus, Δr measures the width of the interval of initial support values for which the outcome entropy remains appreciable.

**Figure 5:**
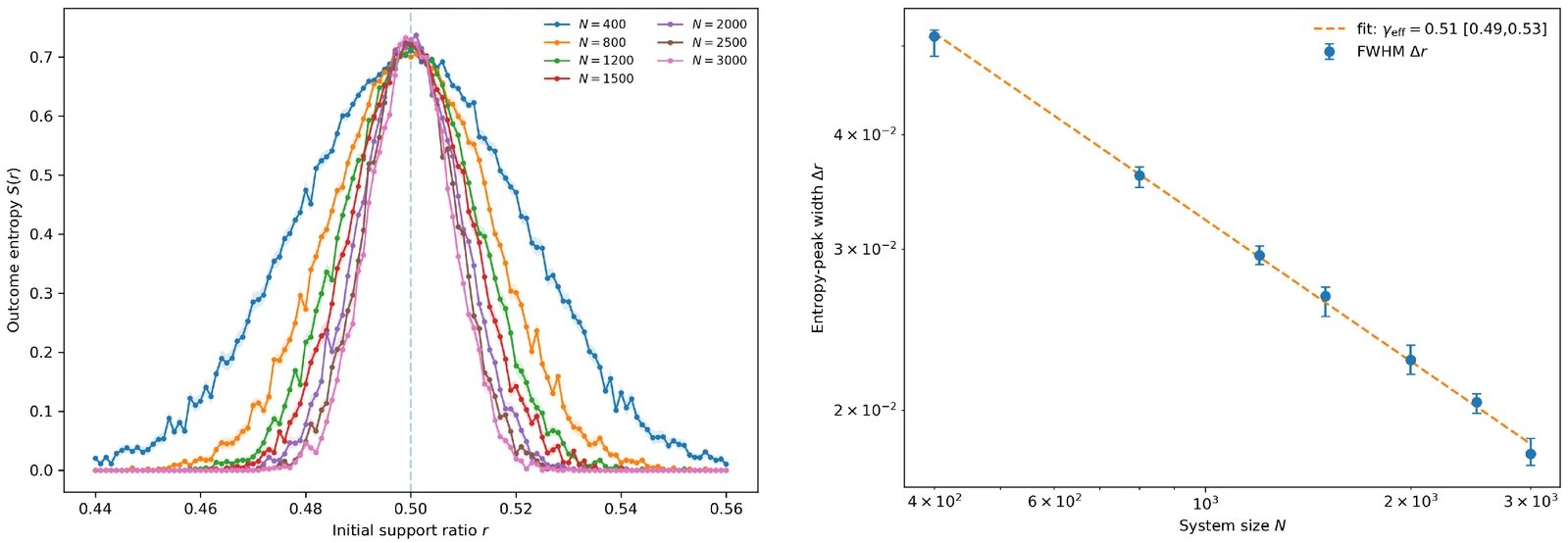
Finite-size sharpening of the high-entropy region. (**Left**) Mean outcome entropy S(r) as a function of *r*, with uncertainty bands across network realizations. As the number of nodes in the network increases, the width of the high-entropy region becomes smaller. (**Right**) The FWHM width Δr of the entropy peak as a function of system size *N*. Error bars are obtained by bootstrap resampling of network-level entropy curves. A log–log fit gives $\Delta r∼N^{-\gamma_{\mathrm{eff}}}$, with $\gamma_{\mathrm{eff}}=0.513$ and a 95% bootstrap confidence interval [0.489,0.526].

Uncertainty in Δr is estimated by bootstrap resampling of the network-level entropy curves. In each bootstrap sample, the *M* network-level curves are resampled with replacement, the mean entropy curve is recomputed, and its FWHM width is extracted. The scaling exponent is then estimated by fitting

(38) $$\Delta r∼N^{-\gamma_{\mathrm{eff}}}$$

on logarithmic axes for each bootstrap sample (see Figure 5). This procedure gives

(39) $$\gamma_{\mathrm{eff}}=0.513,$$

with a 95% bootstrap confidence interval

(40) $$0.489\leq \gamma_{\mathrm{eff}}\leq 0.526.$$

$\gamma_{\mathrm{eff}}$ can be thought of as an effective finite-size exponent for the random-regular topology and parameter range studied. The value is close to the scale expected from fluctuations in the realized initial support. Near r=1/2, binomial fluctuations in the initial support fraction scale as

(41) $$\sigma_{r}=\sqrt{\frac{r(1-r)}{N}}≃\frac{1}{2\sqrt{N}}.$$

Thus, a transition window controlled primarily by finite-size fluctuations in the initial imbalance is naturally expected to narrow approximately as $N^{-1/2}$, with corrections due to topology, correlations, stochastic tie-breaking, and the operational definition of Δr.

**Figure 6:**
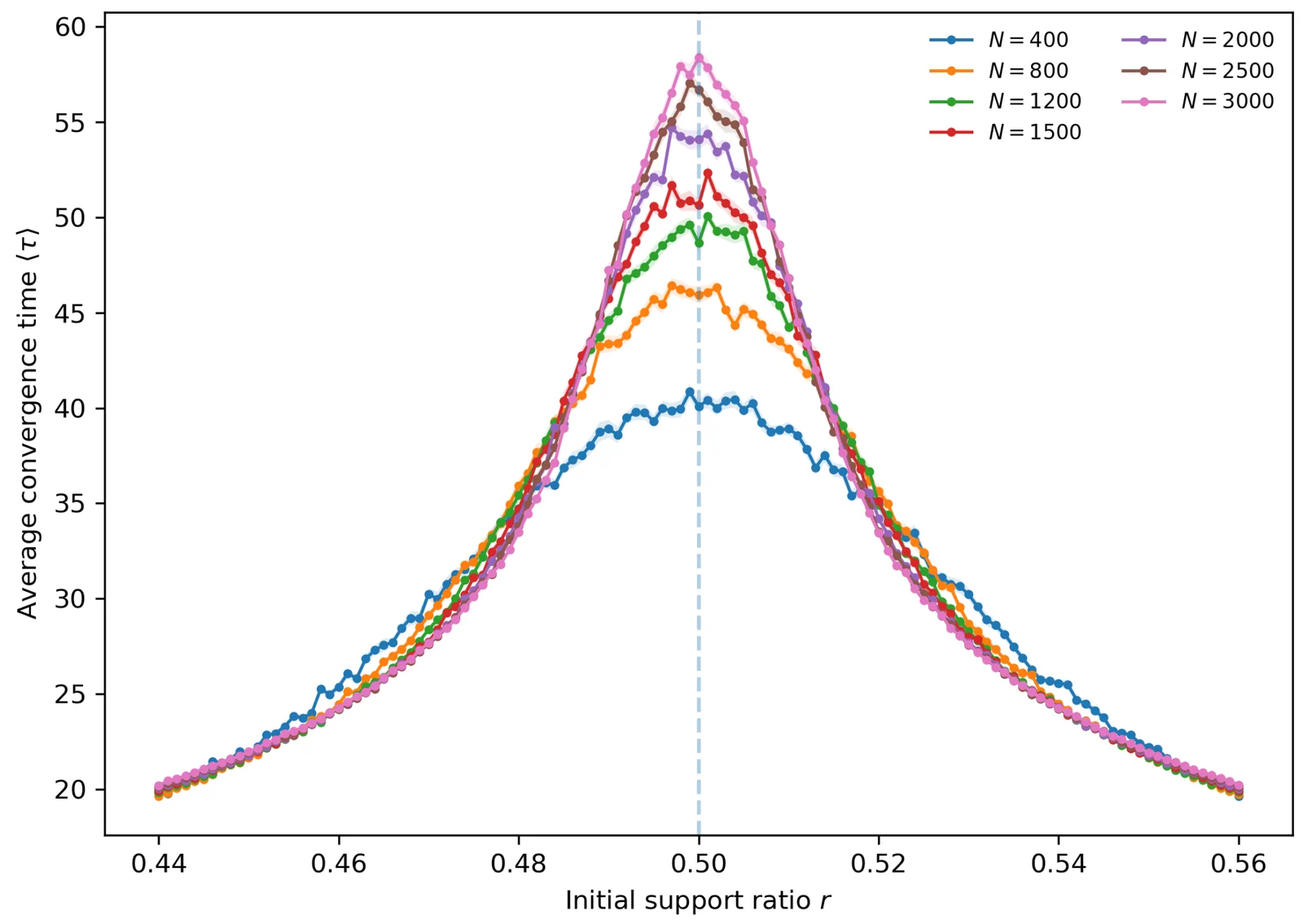
Finite-size sharpening of the convergence time. Mean convergence time 〈τ〉 as a function of *r*, with uncertainty bands across network realizations. The peak near r≃1/2 grows with *N*, consistent with metastable slowing near the boundary between polarized outcomes.

The convergence-time curves show a similar effect (Figure 6). The mean convergence time 〈τ〉 peaks near r≃1/2, and the height of this peak increases with *N*, indicating that trajectories spend longer near the unstable balanced configuration before reaching one of the polarized outcomes.

### 4.5. Topology Dependence of the Transition Region

The finite-size analysis above used independent random 3-regular graphs as a controlled baseline. This choice removes degree heterogeneity and gives a clean setting in which to isolate the effect of antagonistic inter-layer coupling. To test whether the observed behavior is specific to this baseline topology, we repeated the same diagnostic on representative sparse graph ensembles: Erdös–Rényi random graphs, Watts–Strogatz small-world graphs, and Barabási–Albert scale-free graphs.

These simulations used the same antagonistic update rule, independent initialization, coupling J=−1, and time cutoff $T_{\max}=150$. For the topology comparison, we used N=300, M=25 independent network realizations, and R=100 dynamical runs per network realization for each value of *r*.

**Figure 7:**
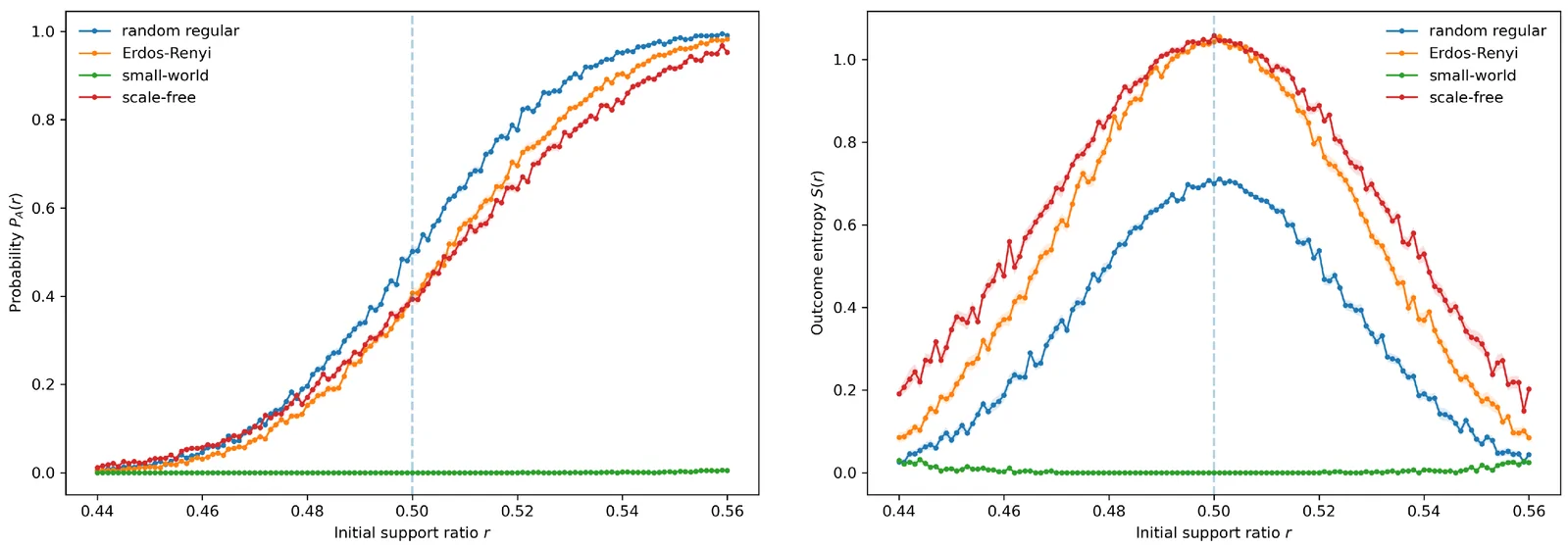
Topology dependence of the transition region. The antagonistic update rule was applied to representative sparse graph ensembles at N=300, with M=25 network realizations and R=100 dynamical runs per network realization. Random-regular graphs show a sharp crossover between A- and B-dominated outcomes and a localized entropy peak. Erdös–Rényi and scale-free graphs show broader transition regions and larger entropy peaks because non-consensus/metastable outcomes contribute near r≃1/2. The small-world ensemble behaves differently: most trajectories remain non-consensus up to $T_{\max}=150$, so $P_{A}$ and $P_{B}$ remain close to zero and the convergence time saturates.

As depicted in Figure 7, the random-regular case shows the cleanest version of the mechanism: the probability $P_{A}\left(r\right)$ crosses from near zero to near one in a narrow interval around r≃1/2, while the outcome entropy has a localized peak. This indicates competition between the two polarized absorbing states, with uncertainty concentrated near equal initial support.

The Erdös–Rényi and scale-free ensembles show the same qualitative A/B competition, but with broader transition regions and larger peak entropies. In these cases, degree fluctuations and structural heterogeneity increase the fraction of runs classified as non-consensus or metastable near the transition. The entropy peak is therefore broader and higher because uncertainty is distributed among three outcome classes: A-dominated, B-dominated, and non-consensus/metastable.

Interestingly, the Watts–Strogatz small-world ensemble behaves in a fundamentally different way over the simulated time window. Instead of rapidly selecting one of the two polarized absorbing states, most runs remain in the non-consensus/metastable class up to $T_{\max}$. Consequently, $P_{A}\left(r\right)$ and $P_{B}\left(r\right)$ remain close to zero over the scanned interval, the convergence time saturates near $T_{\max}$, and the outcome entropy is low because almost all realizations fall into the same non-consensus class. This indicates that clustered small-world structure can trap the system in long-lived patchy configurations that do not resolve into a global A- or B-dominated outcome on the simulated timescale. While the small-world graph supports strong local information circulation through clustered neighborhoods, the sparse long-range shortcuts are not sufficient on the simulated timescale to organize these local domains into one coherent global polarized state.

From these results, we see that antagonistic coupling generically creates competition between incompatible layer states, but the way this competition is resolved depends on topology. Random-regular networks give a sharp two-outcome transition; heterogeneous random and scale-free networks broaden the transition and increase the metastable contribution; clustered small-world networks can suppress global polarized consensus altogether within finite observation time.

### 4.6. Comparison with Cooperative Inter-Layer Coupling

**Figure 8:**
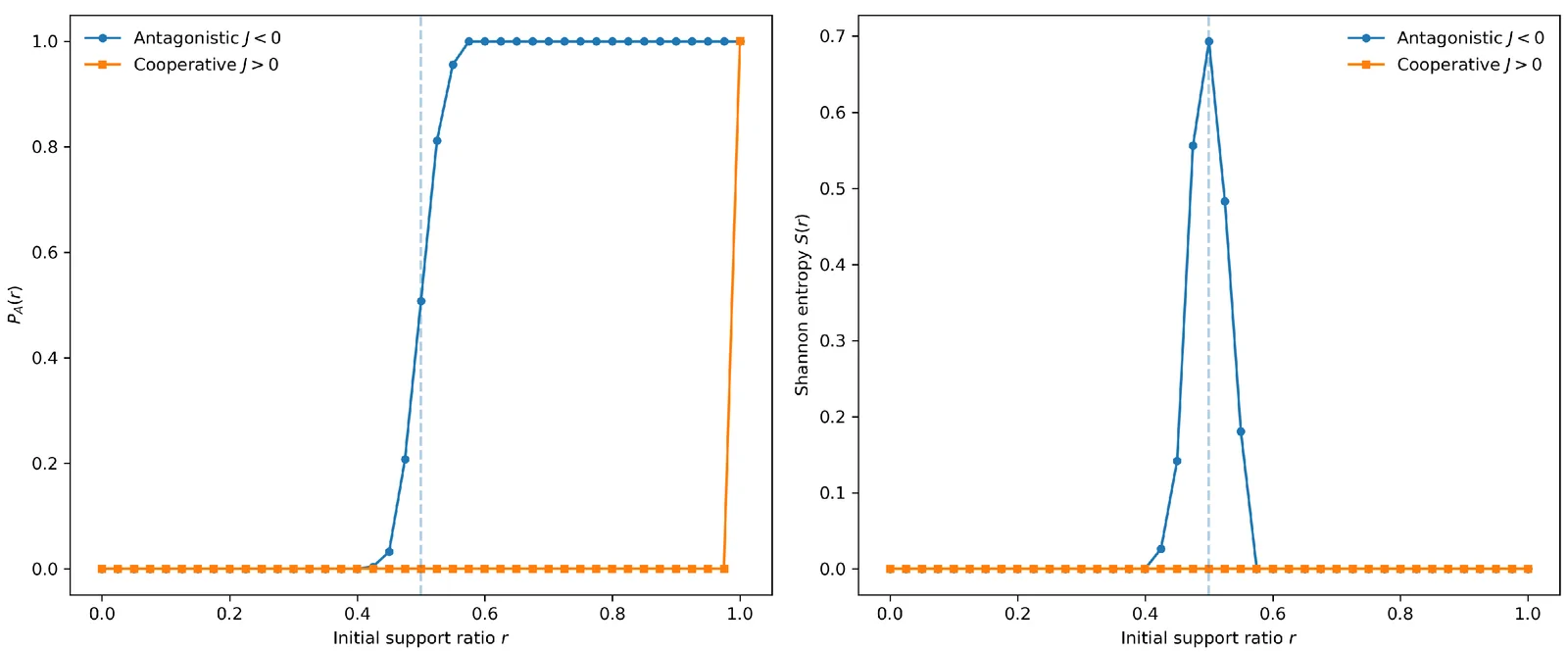
Comparison between antagonistic and cooperative interlayer coupling for N=300 and 41 values of *r*. (**Left**) For cooperative coupling J>0, the system rapidly converges to symmetric consensus states with $m_{A}\approx m_{B}\approx \pm 1$, and the outcome is essentially determined by the sign of the initial bias. (**Right**) Corresponding entropy profiles S(r): a pronounced peak for J<0 indicates a narrow band of highly unpredictable initial conditions, while S(r) remains close to zero and nearly flat for J>0, highlighting the crucial role of antagonistic coupling in generating metastability and outcome uncertainty.

For completeness, we compare the antagonistic case J<0 with cooperative coupling J>0 (Figure 8). In the cooperative regime, the inter-layer term reinforces alignment between the two layers. The stable outcomes are then symmetric consensus states with $m_{A}≃m_{B}$, rather than polarized states with $m_{A}≃-m_{B}$.

Numerically, cooperative coupling suppresses the high-entropy region observed in the antagonistic case. The outcome is more directly determined by the initial bias, convergence is faster, and the entropy profile remains low compared with the antagonistic case. This contrast shows that the entropy peak is not a generic consequence of synchronous majority-rule updating alone. It is generated by the frustration between intra-layer conformity and antagonistic inter-layer inhibition.

## 5. Empirical Case Study: Electoral Entropy Landscape

In the following, we will consider an empirical case study that presents signatures of the antagonistic mechanisms identified in our model. The county-level election outcomes of the 2024 U.S. presidential election are shaped by many factors, including demographic composition, turnout, geography, campaign effects, media exposure, economic variables, and historical voting patterns [6,33,34]. Nevertheless, empirical vote-share and entropy distributions display the diagnostic signatures that are consistent with the antagonistic multiplex model, namely bimodality, low-entropy polarized basins, and a localized high-entropy competitive region.

### 5.1. Data and Pre-Processing

We analyze publicly available county-level returns from the 2024 U.S. presidential election. We used the 2024 county-level U.S. presidential election results from McGovern et al.’s GitHub dataset, which compiles results from sources, including Fox News, Politico, the New York Times, Townhall.com, and The Guardian [35].

For each county *i*, we denote by $V_{i}^{\left(A\right)}$ and $V_{i}^{\left(B\right)}$ the total votes for the two major coalitions (for example, Democratic and Republican), and by

(42) $$T_{i}=V_{i}^{\left(A\right)}+V_{i}^{\left(B\right)}$$

their combined two-party turnout. To focus on the competition between the two dominant blocs, we either discard third-party votes or re-normalize the major-party shares, defining the two-party vote fraction

(43) $$p_{i}=\frac{V_{i}^{\left(A\right)}}{T_{i}},\;1-p_{i}=\frac{V_{i}^{\left(B\right)}}{T_{i}}.$$

The scalar $p_{i}\in \left[0,1\right]$ plays the role of an empirical “magnetization” coordinate for county *i*: values near $p_{i}\approx 1$ correspond to strong support for coalition *A*, values near $p_{i}\approx 0$ to strong support for coalition *B*, and values near $p_{i}\approx 0.5$ indicate balanced support.

### 5.2. County-Level Vote-Share Entropy

For each county, we compute the binary Shannon entropy

(44) $$H_{i}=-\left[p_{i}\ln p_{i}+\left(1-p_{i}\right)\ln\left(1-p_{i}\right)\right]$$

in analogy with the entropy of two-outcome consensus states in our model. Counties with highly skewed vote shares ($p_{i}\approx 0$ or 1) have low entropy $H_{i}\approx 0$, indicating near-unanimous support for one coalition and highly predictable outcomes. In contrast, counties with $p_{i}\approx 0.5$ exhibit maximum diversity and unpredictability, with $H_{i}$ approaching its theoretical maximum at $p_{i}=1/2$.

### 5.3. Empirical Entropy Landscape

**Figure 9:**
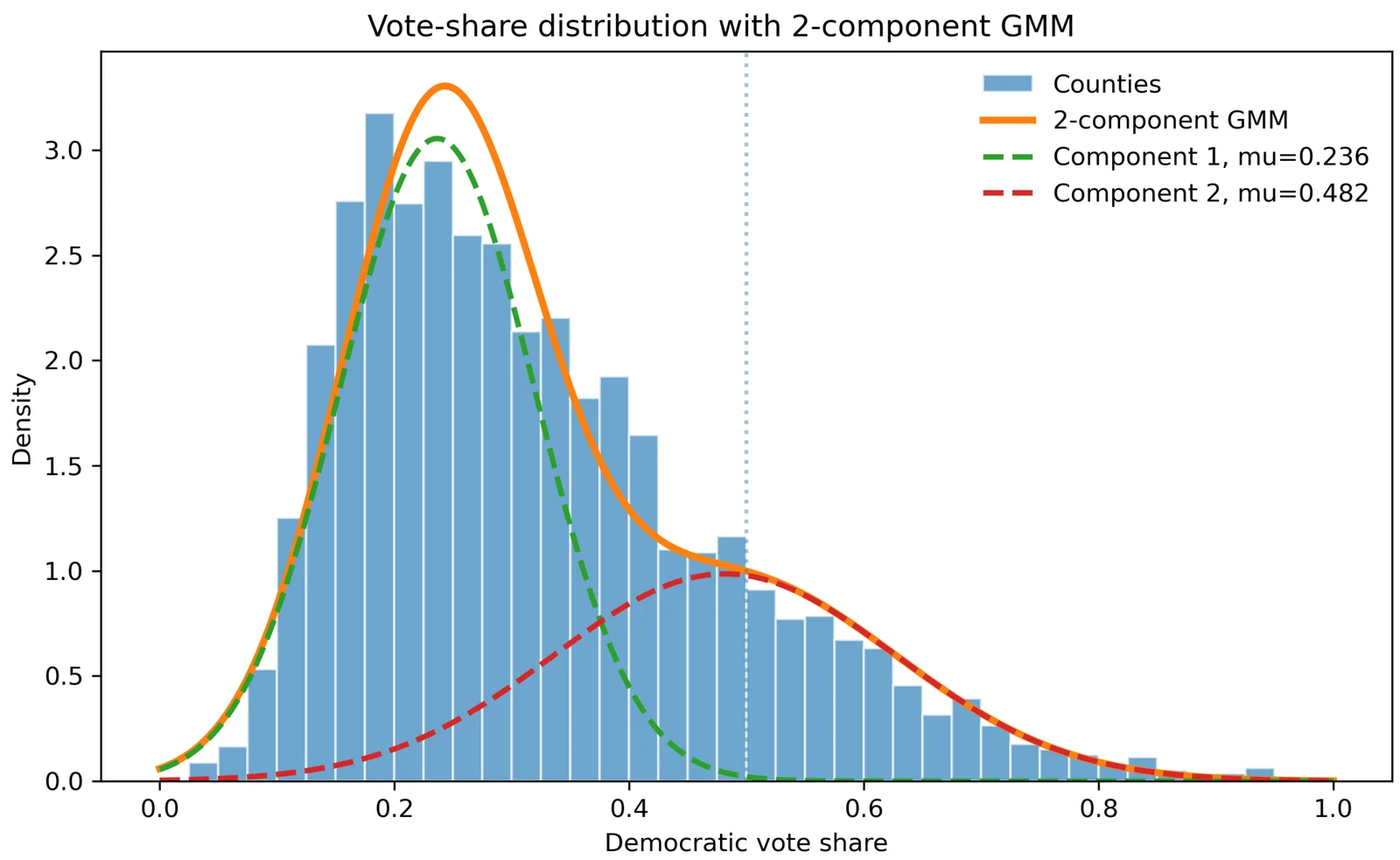
Histogram of the two-party vote fraction $p_{i}$ for coalition A in the 2024 U.S. presidential election across all counties. The distribution is bimodal, with one peak at $p_{i}<0.5$ and another at $p_{i}∼0.5$, reflecting the existence of two broad classes of counties dominated by opposite coalitions. The depletion of probability mass between the Gaussian peaks is consistent with a dual-attractor structure analogous to the polarized consensus states in the antagonistic multiplex model.

The distribution of $\left\{p_{i}\right\}$ across all counties is shown in Figure 9. Under a Gaussian mixture model, the empirical distribution is bimodal, with one peak at $p_{i}<0.5$ and another at $p_{i}∼0.5$. This bimodality reflects the existence of two broad classes of counties:

1. *Partisan Strongholds*, where one coalition dominates the vote share;
2. *Competitive Counties*, where the vote share lies closer to parity.

In the language of our model, the two peaks are analogous to the basins of attraction of the A-dominated and B-dominated consensus states, while the region near $p_{i}∼0.4$ is qualitatively similar to the critical indecision region in the space of initial conditions. The coexistence of two well-separated peaks with a relative depletion near $p_{i}∼0.4$ is consistent with a dual-attractor structure for the underlying social dynamics.

**Figure 10:**
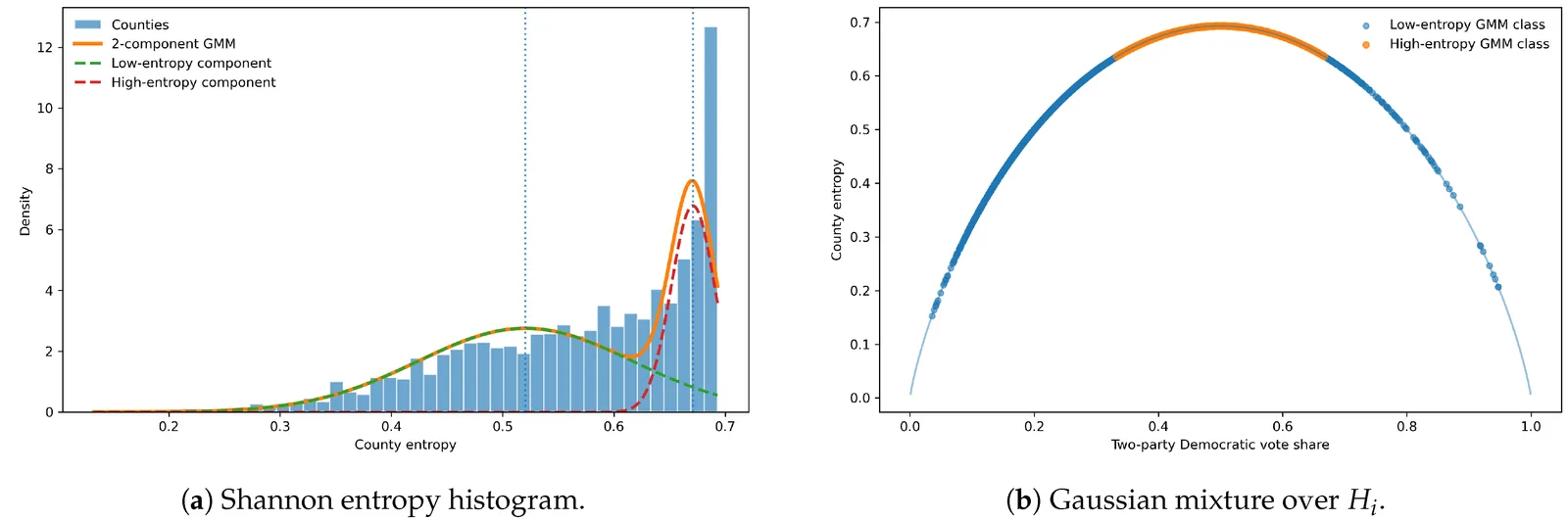
(**a**) Histogram of the Shannon entropy $H_{i}=-\left[p_{i}\ln p_{i}+\left(1-p_{i}\right)\ln\left(1-p_{i}\right)\right]$ across all counties. The distribution shows a substantial mass of low-entropy counties (partisan strongholds) and a secondary concentration of high-entropy counties (swing or competitive counties). (**b**) Fit of a two-component Gaussian mixture model to the one-dimensional distribution of $H_{i}$. The mixture separates counties into low-entropy and high-entropy classes, providing a quantitative characterization of partisan strongholds and swing counties that parallels the low-and high-entropy appearances in the model.

**Figure 11:**
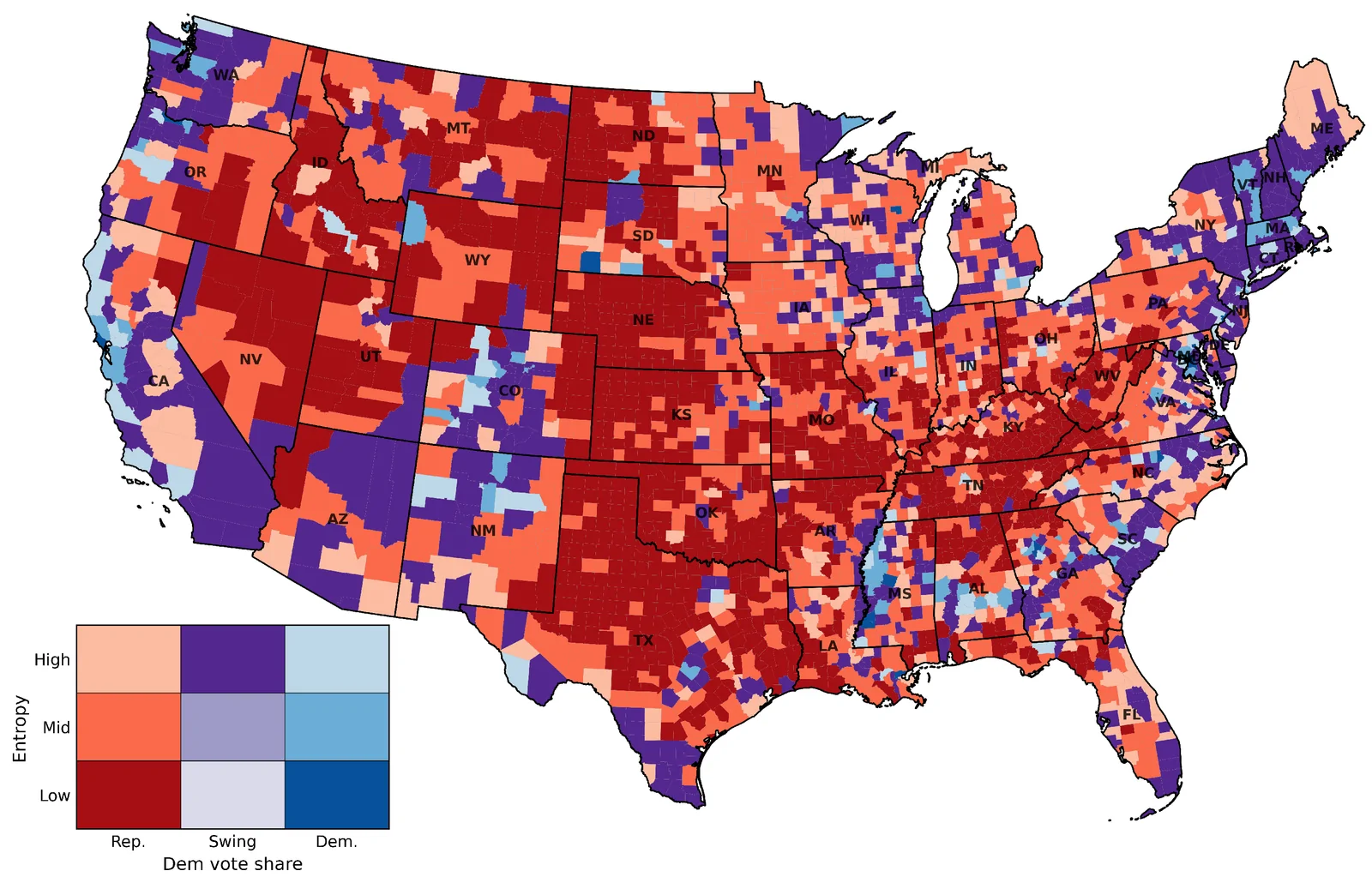
Spatial distribution of county entropy classes in the 2024 U.S. presidential election. Counties are colored according to their most likely Gaussian mixture component (low-entropy vs. high-entropy) based on the fit in Figure 10b. Low-entropy (partisan) counties form large contiguous regions dominated by a single coalition, whereas high-entropy (swing) counties cluster in well-known battleground areas where opposing networks of influence overlap. This spatial pattern mirrors the model picture of a narrow, high-entropy region separating two large polarized consensus basins in the space of initial conditions.

The empirical distribution of $\left\{H_{i}\right\}$ is shown in Figure 10. It is again clearly structured, with a substantial mass of low-entropy counties (partisan strongholds) and a secondary concentration of high-entropy counties (swing or competitive counties). To make this separation more precise, we fit a Gaussian mixture model to the one-dimensional distribution of $H_{i}$, allowing for two components. The resulting mixture assigns each county a posterior probability of belonging to a “low-entropy” or “high-entropy” class. When mapped back onto the geographic layout (Figure 11), these classes reveal spatial structure: high-entropy counties tend to cluster in well-known battleground regions, while low-entropy counties dominate in regions with entrenched partisan identities.

### 5.4. Mapping Theory to Data

The structure revealed by the entropy landscape shares three important features with the theoretical picture developed in the preceding sections:

1. The two peaks in the vote-share distribution (Figure 9) provide an empirical analog of the two polarized basins in the antagonistically coupled multiplex system.
2. The high-entropy subset of counties (Figure 10) plays the role of a competitive or near-critical subset: outcomes are comparatively less predictable and more sensitive to local fluctuations, just as trajectories near r≈0.5 in the model are sensitive to initial support and stochastic tie-breaking.
3. The spatial concentration of high-entropy counties (Figure 11) suggests that regions where opposing networks of influence overlap may be natural empirical analogs of basin-boundary regions in the model.

The qualitative agreement between the model signatures and the empirical election data suggests that frustrated consensus dynamics may provide one possible minimal mechanism for producing similar statistical patterns in polarized competition. The bimodal vote-share distributions observed in U.S. county-level election data are consistent with earlier findings on political polarization by McCarty, Poole, and Rosenthal [33], Gelman et al. [34], Bakshy et al. [5], Flaxman et al. [3], and Brown and Enos [36]. Entropy-based measures have been widely used to quantify diversity and uncertainty in electoral and social systems [37,38]. Gaussian mixture models provide a principled statistical framework for identifying latent regimes in empirical distributions [39,40]. The spatial clustering of high-entropy counties mirrors interface-like regions predicted by frustrated consensus models [41,42,43,44].

## 6. Discussion

The results above show that a minimal antagonistic multiplex extension of majority-rule dynamics is sufficient to generate polarized absorbing outcomes, delayed relaxation near balanced initial support, and a localized peak in outcome entropy. The mechanism is simple: intra-layer majority rule favors local consensus within each layer, while antagonistic inter-layer coupling favors opposite states across corresponding nodes. These two effects cannot be simultaneously reconciled in a neutral way near equal initial support. As a result, the balanced configuration is unstable in the regularized mean-field description, while the two states with opposite layer magnetizations are absorbing in the underlying microscopic dynamics. In terms of entropy, away from r=1/2, the initial bias strongly favors one of the two polarized outcomes, so the final state is highly predictable, and the outcome entropy is low. Near r=1/2, the system lies close to the boundary between the two polarized outcomes. In this region, finite-size fluctuations in the sampled initial condition, stochastic tie-breaking, and the particular network realization can influence which polarized state is reached. The Shannon entropy of outcomes consequently peaks near equal initial support, and the convergence time also increases due to trajectories spending longer near the unstable balanced state before committing to one of the two polarized outcomes.

By performing a finite-size analysis, one can see this structure manifest at the level of numerical simulations. By averaging over independent network realizations, as well as independent dynamical runs, the entropy peak, measured by the FWHM width Δr, is observed to narrow systematically with system size. The estimated scaling

(45) $$\Delta r∼N^{-\gamma_{\mathrm{eff}}},\;\gamma_{\mathrm{eff}}=0.513$$

with a 95% bootstrap confidence interval [0.489,0.526], is very close to the $N^{-1/2}$ scale expected from binomial fluctuations in the initial support imbalance. This supports the interpretation of the transition region as a finite-size uncertainty window around the balanced initial condition. The scope of these results is complemented by the comparisons over different network topologies. The random-regular topology provides a controlled baseline in which the antagonistic mechanism appears as a sharp competition between two polarized absorbing states. In heterogeneous random and scale-free networks, the same competition remains visible, but the transition region broadens, and the non-consensus/metastable class contributes more strongly. In small-world networks, however, the behavior is qualitatively different within the simulated time window: clustered local neighborhoods can sustain long-lived patchy configurations, preventing rapid selection of either global polarized state. Network topology therefore can change the relaxation pathway and the effective outcome structure.

The empirical analysis of the 2024 U.S. county-level presidential election data provides a qualitative comparison with this theoretical picture [35]. The vote-share distribution is bimodal, while the entropy landscape separates low-entropy partisan strongholds from higher-entropy competitive counties. These features parallel the model’s two-attractor structure and its narrow high-entropy transition region, giving a minimal dynamical mechanism with qualitative statistical signatures comparable to those observed in this empirical setting.

Several limitations should be emphasized. First, the model uses binary states and synchronous updates. This is appropriate for a minimal majority-rule model, but real opinion dynamics involve heterogeneous degree distributions, spatial organization, unequal influence, memory, turnout effects, media shocks, demographic structure, and evolving network ties. These are precisely the kinds of factors known to affect dynamics on complex and multilayer networks [18,29,45]. Second, the baseline analysis uses synthetic graph ensembles rather than empirically reconstructed social networks [46,47]. Third, the empirical application uses a single cross-sectional electoral outcome rather than a temporal sequence of dynamical observations. Fourth, the U.S. county-level case is only one empirical setting. A systematic cross-national study would require harmonized subnational election data, comparable administrative units, and consistent two-party or two-bloc definitions across different electoral systems.

These limitations, however, suggest a number of natural extensions. On the theoretical side, one could study asynchronous updates [48], heterogeneous inter-layer coupling [49], weighted networks, more than two layers, or more than two opinion states [21]. It would also be useful to derive sharper analytical predictions for the entropy width and convergence-time scaling, particularly for non-regular graph ensembles. On the network side, hierarchical, modular, or fractal-like topologies may produce multiple relaxation scales, broadened or split entropy peaks, and topology-dependent finite-size exponents [50,51,52].

## 7. Conclusions

We have introduced a minimal antagonistic multiplex model for binary consensus dynamics. Each layer follows local majority-rule updating, while corresponding nodes in different layers interact antagonistically. This combination of intra-layer conformity and inter-layer inhibition produces a frustrated dynamical system with two polarized absorbing outcomes: one in which layer *A* dominates and layer *B* is opposed, and the symmetric outcome with the roles reversed.

A regularized mean-field analysis shows that the balanced state is destabilized through an antisymmetric mode. This identifies the basic mechanism behind the numerical results: near equal initial support, small fluctuations are amplified in opposite directions across the two layers. In the microscopic sign-rule dynamics, this leads to delayed relaxation near the balanced state before one of the two polarized outcomes is reached.

In the network-averaged simulations, the Shannon entropy of outcomes is low when the initial support ratio strongly favors one layer, but develops a localized peak near r=1/2, where the final outcome is most uncertain. The mean convergence time also peaks in this region, indicating metastable slowing near the boundary between polarized outcomes. Using independent network realizations and bootstrap uncertainty estimates, we find that the width of the high-entropy region narrows with system size according to

(46) $$\Delta r∼N^{-\gamma_{\mathrm{eff}}},$$

with

(47) $$\gamma_{\mathrm{eff}}=0.513,\;95\%\;\mathrm{CI}=\left[0.489,0.526\right]$$

This exponent is close to the $N^{-1/2}$ scale expected from finite-size fluctuations in the initial support imbalance. By considering different network topologies, we observed that random and heterogeneous graph ensembles still display competition between polarized outcomes, although the entropy width, convergence time, and metastable fraction depend on topology. The small-world case is especially distinct: clustered local structure can support long-lived non-consensus configurations within the simulated time window, preventing rapid selection of either global polarized state. Thus, topology controls how antagonistic competition is resolved, even when the underlying update rule is unchanged.

Empirical election analysis revealed that county-level vote-share entropy separates low-entropy partisan strongholds from higher-entropy competitive counties, providing a descriptive analog of the model’s distinction between polarized outcomes and high-uncertainty regions. This illustrates that entropy and bimodality can serve as useful diagnostics of polarized competition in empirical multiplex social systems.

Overall, the results show that antagonistic coupling between internally consensus-seeking layers provides a simple mechanism for the coexistence of polarized attractors, metastable delay, and localized outcome uncertainty. Future work will investigate asynchronous updating, heterogeneous coupling strengths, weighted and empirical networks, multi-layer and multi-state extensions, and topology-dependent scaling. Future work should also investigate hierarchical or fractal-like network structures, which may generate multiple relaxation scales, broadened or split entropy peaks, and topology-dependent finite-size behavior.

## Data Availability

The data analyzed in this study is available at the GitHub repository described in [35].
